# Computational design for enantioselective CO_2_ capture: asymmetric frustrated Lewis pairs in epoxide transformations

**DOI:** 10.3762/bjoc.20.224

**Published:** 2024-10-22

**Authors:** Maxime Ferrer, Iñigo Iribarren, Tim Renningholtz, Ibon Alkorta, Cristina Trujillo

**Affiliations:** 1 Instituto de Química Médica (CSIC), Juan de la Cierva, 3, 28006 Madrid, Spain; 2 Technische Universität München (TUM), School of Computation, Information and Technology, D-85748 Garching, Germany; 3 Department of Chemistry, The University of Manchester, Oxford Road, Manchester, M13 9PL, UK; 4 Trinity Biomedical Sciences Institute, School of Chemistry, The University of Dublin, Trinity College, D02 R590 Dublin 2, Ireland

**Keywords:** asymmetric catalysis, carbon dioxide, CO_2_, epoxide, frustrated Lewis pairs (FLPs), volcano plot

## Abstract

Carbon capture and utilisation (CCU) technologies offer a compelling strategy to mitigate rising atmospheric carbon dioxide levels. Despite extensive research on the CO_2_ insertion into epoxides to form cyclic carbonates, the stereochemical implications of this reaction have been largely overlooked, despite the prevalence of racemic epoxide solutions. This study introduces an in silico approach to design asymmetric frustrated Lewis pairs (FLPs) aimed at controlling reaction stereochemistry. Four FLP scaffolds, incorporating diverse Lewis acids (LA), Lewis bases (LB), and substituents, were assessed via volcano plot analysis to identify the most promising catalysts. By strategically modifying LB substituents to induce asymmetry, a stereoselective catalytic scaffold was developed, favouring one enantiomer from both epoxide enantiomers. This work advances the in silico design of FLPs, highlighting their potential as asymmetric CCU catalysts with implications for optimising catalyst efficiency and selectivity in sustainable chemistry applications.

## Introduction

The field of frustrated Lewis pairs (FLPs) has flourished since their seminal discovery in 2006 by Stephan and colleagues [1]. These compounds, which feature a Lewis acid (LA) and a Lewis base (LB), whose interaction is hindered by bulky substituents or chain strain, have garnered significant attention. Initially explored for their ability to trap small molecules [2–3], such as H_2_ [4], CO_2_ [5–7], N_2_O [8–9], and alkenes [10–11], they have since found applications in catalysis [12–13].

Among the first catalytic uses of FLPs were the hydrogenation of unsaturated compounds [12,14] and the reduction of CO_2_ using H_2_ as a reductant [7,15–17]. FLPs have become an attractive avenue for the reduction of CO_2_, particularly given the increasing levels of CO_2_ in the atmosphere. However, challenges persist in understanding and optimising the reactivity of these systems.

One significant obstacle is the tendency for CO_2_ to react preferentially with FLPs over H_2_. As such, the design of FLPs that prioritise the capture of H_2_ over CO_2_ becomes crucial for effective CO_2_ reduction [7]. Additionally, the strength of the interaction between the catalyst and the resulting system after hydride transfer presents a limitation. The formation of a robust LA–oxygen interaction may impede proton transfer to the basic oxygen atom. These limitations suggest that a more viable approach to employing FLPs as catalysts for CO_2_-related reactions could involve their use in CO_2_ activation [7,18–19]. In particular, the capture of CO_2_ by FLPs enhances the electrophilicity of the CO_2_ carbon atom and the nucleophilicity of one of the CO_2_ oxygen atoms [6–7].

Carbon capture and utilisation (CCU) technologies involve the extraction of CO_2_ from the atmosphere of the Earth to generate value-added chemicals, which can serve as platform chemicals in other chemical processes [20–21]. This is achieved by inserting CO_2_ as a C1 building block into readily available substrates such as epoxides, resulting in the formation of polycarbonates or monomeric cyclic carbonates [22]. Depending on the substitution pattern in the epoxide, a chiral centre is present in the product.

The insertion of CO_2_ into epoxides has been the subject of numerous studies, but the stereochemical aspects of this reaction, particularly through the use of FLP catalysts, have been largely overlooked, despite the prevalence of racemic epoxide solutions. Only one study has addressed the asymmetric insertion of CO_2_ into propylene oxide (PO) using a transition-metal catalyst [23–25]. Therefore, the stereochemical aspects of CO_2_ insertion into PO enabled by FLP catalysts should be investigated.

**Scheme 1:**
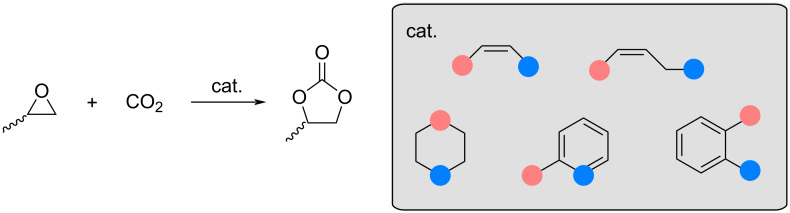
Reaction between propylene oxide (PO) and CO_2_ and the five catalyst scaffolds under study. The position of the LB along with an appropriate number of substituents is indicated by blue dots and that of the LA by pink dots.

To the best of our knowledge, only one paper has proposed an asymmetric approach to this reaction using a metal-based catalyst [23]. However, our approach differs significantly and seeks to explore new possibilities in this area. Herein, the present study focusses on the asymmetric insertion of CO_2_ into PO using asymmetric FLPs as catalysts. Initially, five FLP scaffolds with different substituents, LA and LB, were tested, resulting in a total of 53 potential catalysts (Scheme 1). The most promising catalyst scaffolds for the reaction under study were identified by volcano plot analysis [26–27]. Inspired by the asymmetric oxazoline synthesised by Gao et al. [28], and guided by the volcano plot results, modifications to these FLP scaffolds facilitated the development of an asymmetric FLP and consequently an asymmetric catalyst. The subsequent study explores the asymmetric insertion of CO_2_ into chiral PO catalysed by the proposed in silico designed catalyst.

## Computational Details

During the benchmark to choose the best catalyst, the reported geometries were optimised with the Gaussian16 quantum chemical software package [29], using the B3LYP density functional [30–31] along with the Grimme dispersion correction including Becke and Johnson damping D3(BJ) [32–34] and the def2-TZVP basis set [35]. Harmonic frequencies were computed at the optimisation level to confirm that the relaxed structures correspond to local minima (no imaginary frequencies) or transition states (one imaginary frequency). The reaction simulations were run in chloroform using the “Solvation Model based on Density” (SMD) [36] at 273.0 K to reproduce the most commonly used experimental conditions [37–39].

When considering asymmetry, it was necessary to include large substituents on the catalyst to induce steric hindrance. These modifications increase the size of the asymmetric catalysts. Thus, the calculations presented in subsection “Asymmetric catalysis” were optimised at the B3LYP-D3(BJ)/def2-SVP computational level. Single point energy calculations on the optimised structures were run at the B3LYP-D3(BJ)/def2-TZVP level to obtain more accurate electronic energies. The reported free energies in this section correspond to the sum of the triple-zeta electronic energy and the free energy correction at double-zeta.

The kinetics of some reactions were calculated, applying the transition state theory [40]. Within this theory, the rate constant of an elementary reaction with the free energy barrier Δ*G*^‡^ is given by Equation 1,


[1] $$k=\frac{k_{\text{B}}T}{h}e^{\frac{-\Delta G^{‡}}{RT}},$$

where *k* is the rate constant in s^−1^, *k*_B_ is the Boltzmann constant, *T* is the temperature in Kelvin, *h* is the Planck constant, and *R* is the gas constant.

The enantiomeric excess (%ee) was calculated using Equation 2 [41]. *k*_fav_ stands for the kinetic rate constant of the most favourable process, and *k*_defav_ stands for the rate constant of the less favourable process.


[2] $$\%\text{ee}=\frac{k_{\text{fav}}-k_{\text{defav}}}{k_{\text{fav}}+k_{\text{defav}}}.$$

During the study, it will be observed that several transition states (TSs) can lead to the same product. As there is no possible interconversion between the reactant states, the different reactions will be considered independent, and it will be necessary to use an effective rate constant (*k*_eff_). The definition given by Williams will be used (Equation 3, [42]):


[3] $$k_{\text{eff}}=\sum_{j}^{N_{\text{TS}}}e^{-\Delta^{‡}G_{j}/RT}.$$

The proton affinity (PA) [43] of the LB and the fluoride ion affinity (FIA) [44] of the LA of a given FLP are generally used to rationalise the FLP reactivity observed [45–46]. Thus, PA and FIA of the different scaffolds considered were calculated using Equation 4 and Equation 5, respectively, where H(A) stands for the enthalpy of the FLP, H(H^+^) for the enthalpy of the proton, H(F^−^) for the enthalpy of the fluoride ion, and H([A-H^+^]) and H([A-F^−^]) for the enthalpies of the complexes formed between the FLP and a proton and a fluoride ion, respectively.


[4]

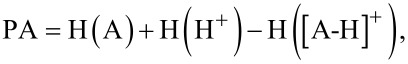




[5]

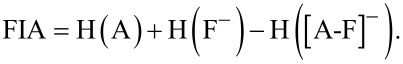



Volcanic 1.3.3, a Python package for the NaviCat platform, was used to generate 3D volcano plots, facilitating the identification of the most appropriate catalyst for the coupling reaction being considered [27].

### Volcano plots

**Figure 1:**
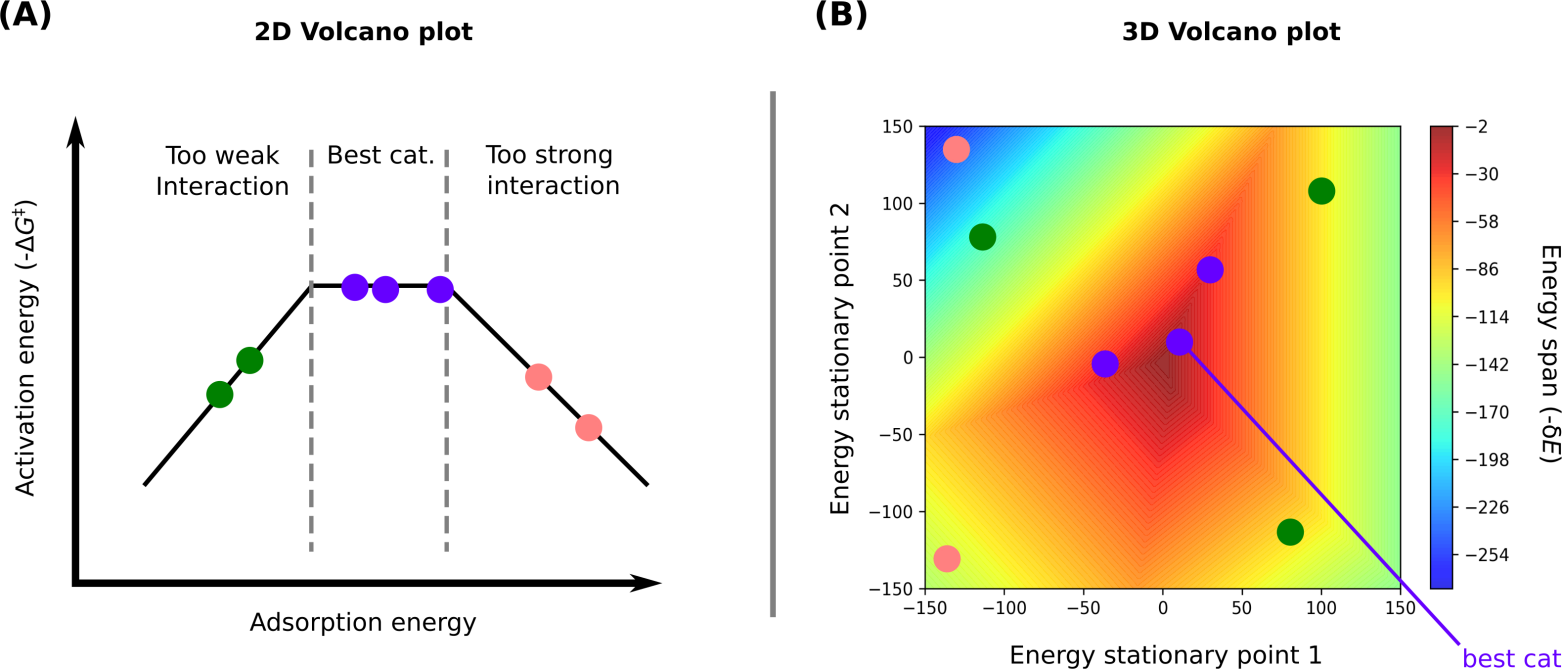
Schematic representation of an (A) 2D and a (B) 3D volcano plot. The abbreviation “cat.” stands for catalyst.

Volcano plots are a visualisation of the Sabatier principle [47], a qualitative concept originating in heterogeneous catalysis for assessing the performance of different catalysts. According to this principle, an ideal catalyst interacts with reactants neither strongly nor weakly. This idea is visualised in volcano plots, where a metric of the catalyst performance, for example, the reaction rate, is displayed as a function of the catalyst–substrate interaction, for example, the adsorption energy when considering a heterogeneous catalyst (Figure 1A). The resulting plot exhibits a volcano-like shape consisting of at least two slopes with the best-performing catalysts located at the top. The top of the volcano plot corresponds to the scenario where the catalyst exhibits high catalytic activity, as it achieves an optimal balance in binding to the reactants, neither too strongly nor too weakly (Figure 1A, purple points). The catalysts with binding energies lower than the catalysts at the top show lower catalytic activity due to insufficient activation of the substrate (Figure 1A, green points). Conversely, catalysts that bind too strongly impede the detachment of the catalyst–reactant complex, thereby reducing the catalyst turnover (Figure 1A, pink points) [26–27].

The previous plots are effective for metal-based catalysts and relatively simple catalytic reactions; however, they fall short when reactions involve multiple steps and independent activation barriers. In this paper, instead of focusing solely on the activation energy, the energy span of the catalytic reaction (δ*E*) is considered. King et al. [48] introduced the concept of the energy span of a simulated catalytic cycle by defining it as the difference between the highest and lowest free energy stationary points [49–50]. More precisely, the energy span can be defined using Equation 6, where *T_i_* is the energy of the rate-limiting TS, *I_j_* the energy of the most populated intermediate, and δ*G_i,j_* a correction that accounts for the cyclic nature of the catalytic cycle [26]:


[6] $$\delta E=\max_{i,j}\left(T_{i}-I_{j}\right)+\delta G_{i,j}.$$

The energy span is a crucial parameter as it directly correlates with the turnover frequency (TOF) of the catalytic reaction (Equation 7). A flatter energy profile, indicated by a δ*E* value closer to zero, signifies more efficient catalysis:


[7] $$\text{TOF}=\frac{k_{\text{B}}T}{h}e^{\frac{-\delta E}{RT}}.$$

In this work, to achieve better correlations between the energy span and the system energies, two energies were used (Figure 1B). The volcanic program employs a multivariate linear regression process. Considering a reaction with six stationary points (REACTANTS, E1, TS2, E2, TS3, and PRODUCTS), which can be catalysed by *n* potential catalysts, the program calculates the correlation between the energy span and all possible pairs of stationary points for the *n* catalysts. For instance, it determines a function such as E1 = *f*(E2, TS3). The quality of these correlations is assessed through the square of the Pearson coefficient. The pair of stationary points has to correlate with the energies of the six stationary points previously presented. The quality of the pair considered to describe well the energies of the catalytic reaction is, thus, obtained by taking the mean value of *R*^2^ of the six correlations. The pair with the largest mean *R*^2^, is considered as the optimal pair of stationary points; it is then used to predict the energy span, resulting in a 2D contour plot (Figure 1B). The *x* axis represents the free energy of the first stationary point of the selected pair and the *y* axis represents the free energy of the other stationary point of the pair. According to the volcano plots, the best theoretically predicted catalysts are those nearest to the lowest predicted δ*E* values, depicted by the purple points in Figure 1B.

## Results and Discussion

The following nomenclature will be used during the volcano plot analysis: FX_LBLA_S1_S2 where X is the label of the family (1, 2, 3, 5, or 6), LB is the Lewis base considered (N or P), LA is the Lewis acid (in this particular study only B), S1 is the substituent on the LB, and S2 is the substituent on the LA.

### Capture of CO_2_ and PO by an FLP

#### Chemoselectivity

Our investigations began by examining the uncatalysed coupling reaction between CO_2_ and PO (Scheme 1), which exhibits a calculated activation barrier (Δ*G*^‡^) greater than 55 kcal·mol^−1^ (Figure S6, Supporting Information File 1). Therefore, in order to observe the coupling between these two moieties under standard conditions, the presence of a catalyst is necessary. In the literature, metal-based and organocatalysts have been reported as efficient catalysts for this reaction [24–25]. As noted previously [51–52], the reaction depicted in Scheme 1 can proceed via two distinct mechanisms.

**Scheme 2:**
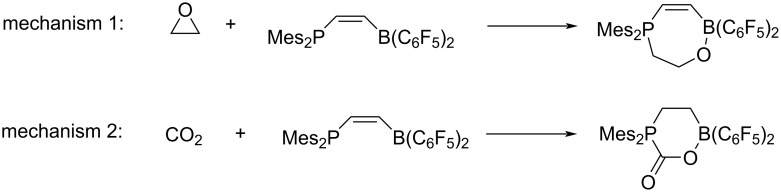
Capture reactions of CO_2_ or an epoxide by FLP.

In the first mechanism, the catalyst initiates epoxide opening, followed by CO_2_ insertion. The second mechanism suggests that CO_2_ activation by the catalyst precedes its transfer to the epoxide. To determine the more feasible mechanism, a comprehensive investigation of both possibilities was conducted. To determine the most probable mechanism within our system, the capture of CO_2_ and a symmetric epoxide (E) using the FLP proposed by Stephan et al. [37] was evaluated (Scheme 2). A symmetric epoxide was chosen to avoid addressing asymmetry concerns at this stage. The capture exhibiting the lowest activation barrier was considered the first step of the coupling reaction for the remainder of the study. The free-energy profiles of both capture processes are depicted in Figure S1 (Supporting Information File 1). Notably, the CO_2_ capture exhibits a lower activation barrier compared to the capture of epoxide (+10.0 kcal·mol^−1^ vs +30.0 kcal·mol^−1^). Using transition state theory [40] as expressed in Equation 1, the rate constants were calculated for binding to either molecule at 273.0 K, resulting in *k*_1_ = 5.47·10^4^ s^−1^ for capturing CO_2_ and *k*_2_ = 4.85·10^−12^ s^−1^ for capturing the epoxide. Despite the FLP–CO_2_ adduct being less thermodynamically stable than the FLP–epoxide adduct (−10.1 kcal·mol^−1^ vs −44.8 kcal·mol^−1^), the lower activation barrier for the capture of CO_2_ and the temperature considered (273.0 K) suggest a kinetically controlled reaction. To further shift the chemical equilibrium toward CO_2_ capture, increasing steric hindrance at the epoxide was explored by introducing bulky substituents into the scaffold. This resulted in an increase in activation barriers for adduct formation. Including a methyl group, for instance, increased the barrier by 1.4 kcal·mol^−1^, a phenyl group by 1.7 kcal·mol^−1^, and a *tert*-butyl group by more than 2 kcal·mol^−1^ (Table S1, Supporting Information File 1). This observation is consistent with reports in the literature [24,53–56]. Based on this initial study, it can be concluded that the mechanism for our system proceeds according to mechanism two. The following simulations were performed on this conclusion.

#### Regioselectivity

**Figure 2:**
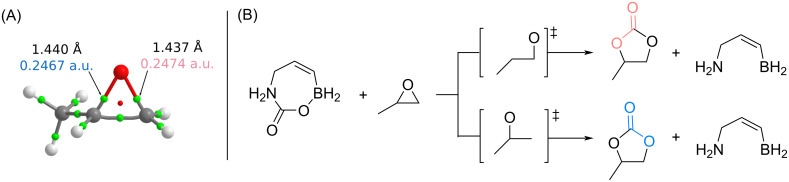
(A) Structure of PO annotated with the C–O bond distances and electron densities at the BCPs. BCPs are indicated by green spheres and the ring critical point by a red sphere. (B) Schematic representation of the two possible ring-opening reactions of PO in the presence of activated CO_2_.

PO exhibits two distinct electrophilic sites, which can be subject to nucleophilic attack (Figure 2B). Thus, the regioselectivity of the CO_2_ insertion into PO must be addressed as part of the full mechanistic investigation. The compound 3-boryl-2-propen-1-amine is now considered as the catalyst (Figure 2B). As observed in Figure 2A, the bond length and electron density at the bond critical point (BCP) difference are minimal and do not conclusively suggest that one bond will be broken more easily than the other. Therefore, both scenarios will be explored to see if the coupling reaction could proceed more easily by breaking the O–CH(CH_3_) bond rather than the O–CH_2_ bond.

Based on our investigations, the opening of PO with activated CO_2_ was found to proceed through two transition states. The calculations showed that the breaking of the O–CH(CH_3_) bond was more kinetically favourable, with a TS 7.6 kcal·mol^−1^ lower in free energy than the corresponding TS for breaking the O–CH_2_ bond. The electron-donating nature of the methyl group facilitates a greater stabilisation of the intermediary positive charge at the central carbon compared to the hydrogen after bond-breaking at the terminal carbon, thereby reducing the activation barrier.

Henceforth, in this paper, the optimised TSs will consistently represent the breaking of the O–CH(CH_3_) bond. Additionally, the (*S*)-epoxide enantiomer was employed consistently.

### Symmetric FLP scaffolds – achiral environment

**Figure 3:**
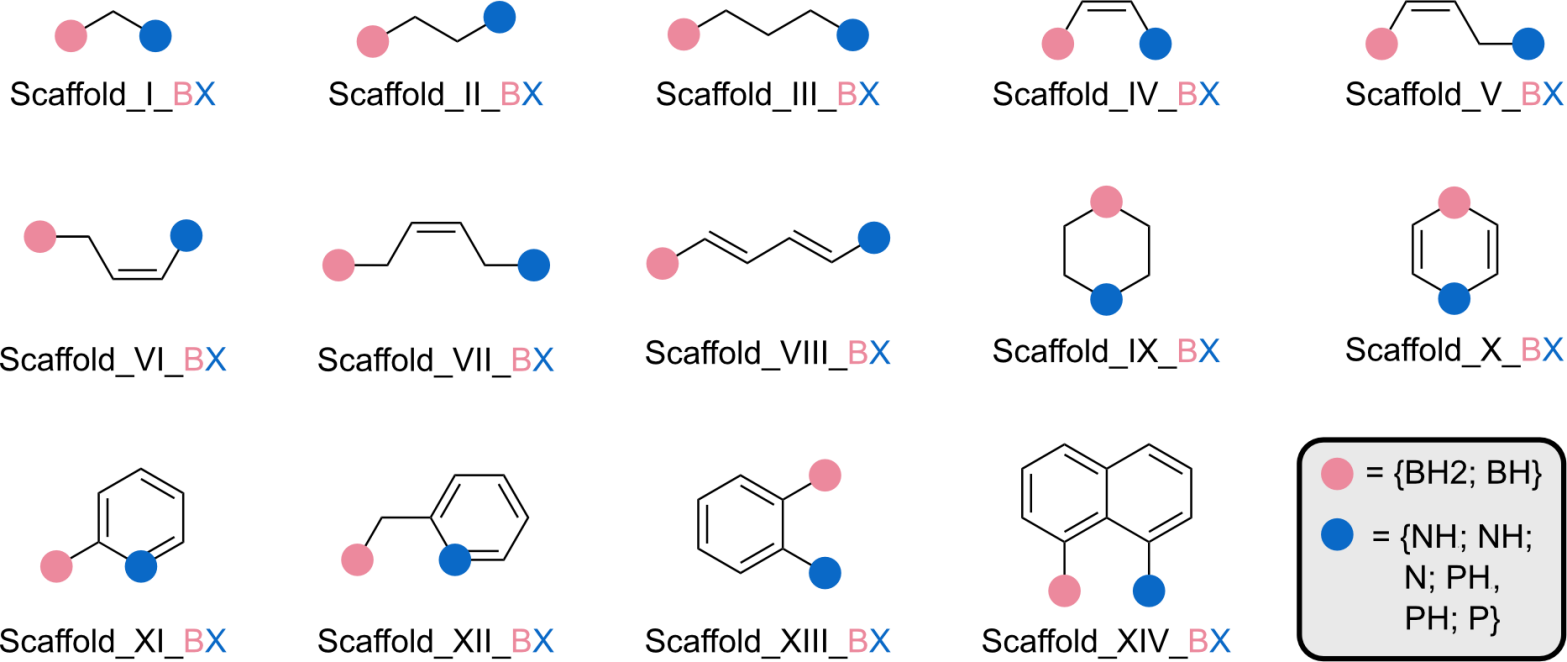
Symmetric FLP scaffolds considered in the first study. X denotes N or P.

Following the initial exploration and preliminary results, our attention shifted toward the identification of a suitable catalyst. Drawing inspiration from the literature, fourteen FLP scaffolds have been evaluated (Figure 3), focussing specifically on N/B and P/B FLPs because of their widespread application in this field, especially considering the initial step involving CO_2_ capture [6,12,21].

#### Selection of the scaffolds and substituents

Volcano plots were introduced to find the most efficient catalyst for a given reaction [26,57]. They are a valuable tool for the in silico design of catalysts [27,58]. Volcano plot analysis requires a set of reactions that follow the same mechanism but whose stationary points possess different energies. Generally, the larger the differences in energy between the stationary points, the better the exploration of the catalytic space.

**Figure 4:**
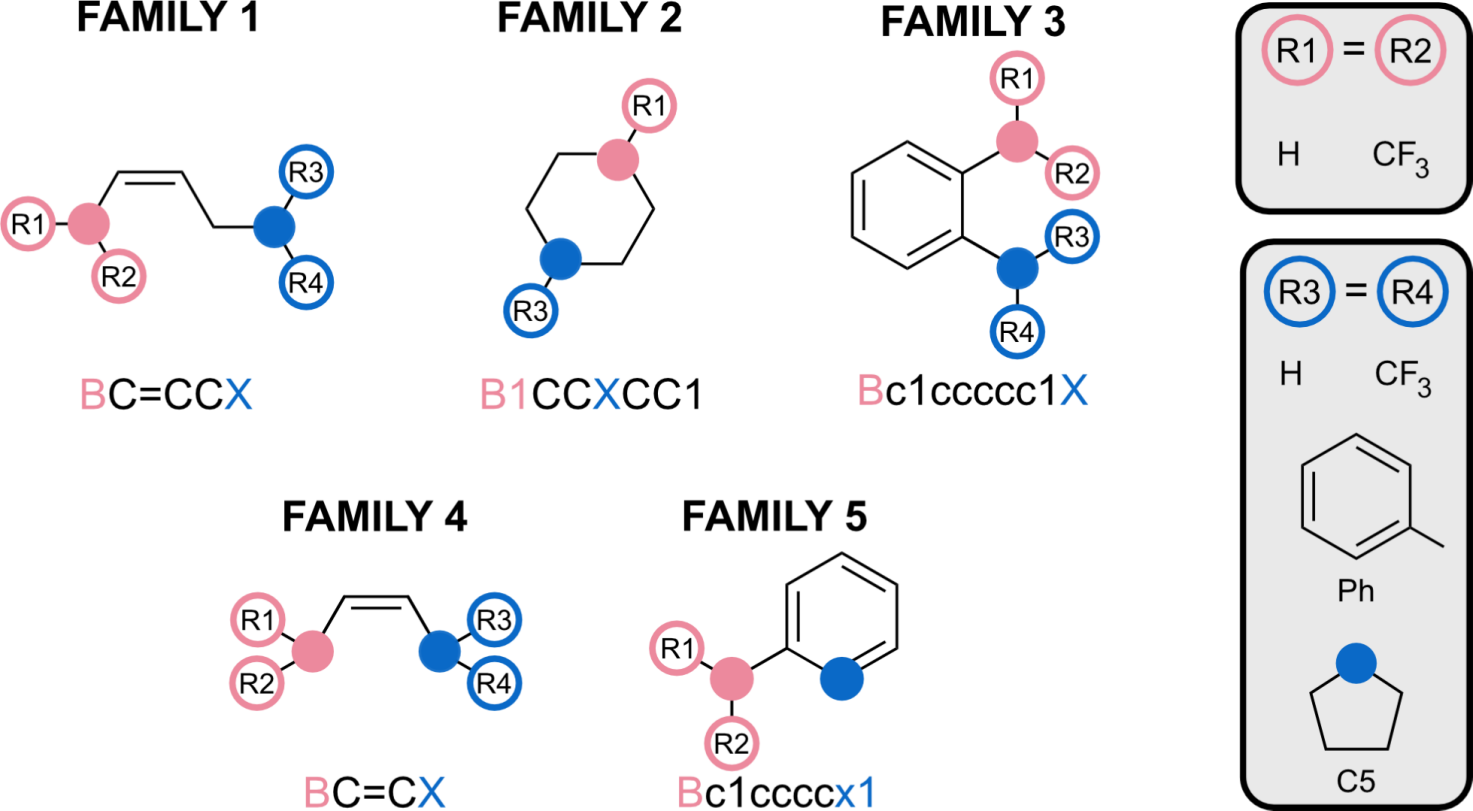
Subset of FLP scaffolds considered in the catalyst optimisation study. Substituents and labels are indicated.

To determine the set of scaffolds to be used for volcano plot analysis, the CO_2_–FLP adduct of each of the fourteen scaffolds was optimised (Figure 3). Based on the stability of the optimised adducts, families can be selected to cover a wide energy range. The obtained free energies of formation are presented in Figure S2 (Supporting Information File 1). The stabilities of the N/B adducts range from −9 to +48 kcal·mol^−1^, while the P/B adducts vary from +10 to +36 kcal·mol^−1^. The reliability of a volcano plot is based on an extensive exploration of the energetic space. Thus, because of the larger variation in the energy of the CO_2_–FLP adducts employing N/B FLPs, it was decided to choose systems based on FLPs with N/B. It is expected that the large energy range of the CO_2_–FLP adducts will propagate along the reaction path, enabling appropriate energy exploration. The scaffolds V_BX (family 1, Δ*G*(adduct) = −0.4 kcal·mol^−1^), IX_BX (family 2, Δ*G*(adduct) = −2.1 kcal·mol^−1^), XIII_BX (family 3, Δ*G*(adduct) = +3.0 kcal·mol^−1^), IV_BX (family 4, Δ*G*(adduct) = +15.4 kcal·mol^−1^), and XI_BX (family 5, Δ*G*(adduct) = −19.5 kcal·mol^−1^) were selected for further investigation (Figure 4). This selection allows us to obtain free energy differences of 35 kcal·mol^−1^ already in the adduct stationary point.

After selecting the scaffolds to work with, the next step is to choose substituents for placement on the LA and LB positions. These substituents will have two main effects on the FLP. First, they will alter the Lewis acidity and basicity of the LA and LB centres, respectively; second, they may induce steric hindrance. The first effect is perhaps the most intriguing to consider, as the acidity and basicity of the LA/LB centres are indicative of the FLP’s reactivity [45–46]. Thus, substituents must be selected to ensure a broad spectrum of acidity and basicity of the LA and LB. Different methods for determining these properties have been described in the literature. Because of their easy computation, the proton affinity [43] and fluoride ion affinity [44] were selected to compute the basicity and acidity of the systems considered. By selecting the substituents presented in Figure 4, FIAs spanning a range of 60 kcal·mol^−1^ and PAs spanning a range of 48 kcal·mol^−1^ were obtained (Figure S3, Supporting Information File 1). All the structures studied exhibit the classical FLP characteristics, except for some systems that can be considered as “masked FLPs” (Table S4, Supporting Information File 1).

#### Possible mechanisms

**Figure 5:**
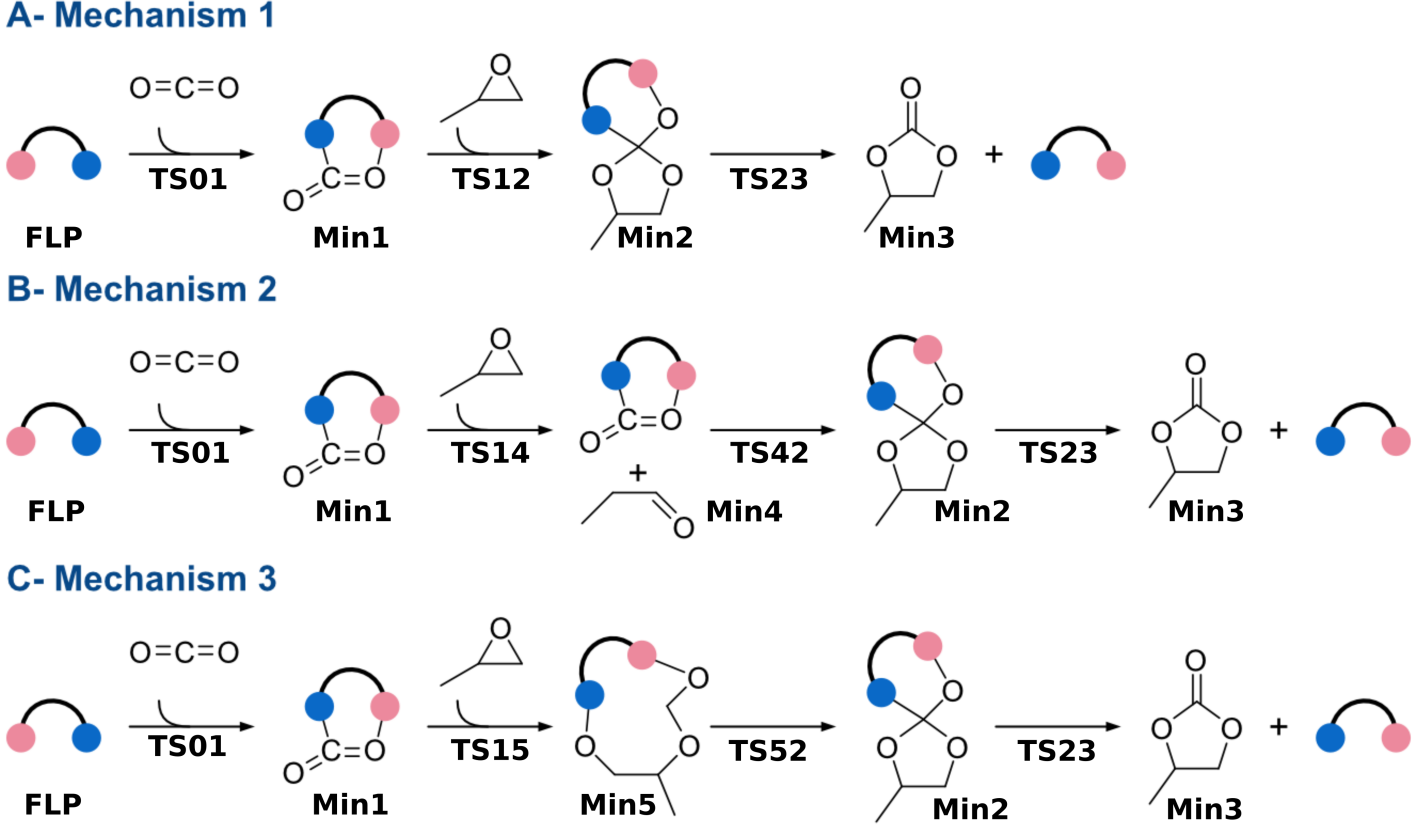
Coupling reaction between PO and CO_2_. Depending on the catalyst considered, the reaction follows mechanism 1, 2, or 3.

As established in the previous section. the general mechanism of the coupling reaction proceeds through three steps: (1) capture of CO_2_, (2) opening of PO and addition of the activated CO_2_, and (3) liberation of the product (Figure 5).

During the study of the selected catalysts (Figure 4), it was observed that, depending on the catalyst considered, the reaction followed a different mechanism, that is, mechanism 1, 2, or 3 (Figure 5). Mechanism 1 (Figure 5A), comprises three steps. First, CO_2_ is captured by FLP (TS01), and subsequently activated CO_2_ is inserted into the epoxide (TS12). TS12 corresponds to the concerted opening of PO and the insertion of CO_2_. The product is later released via TS23. This mechanism is followed by 40% of the catalysed reactions studied. Mechanism 2 (Figure 5B) contains an additional step. In this mechanism, the epoxide is first isomerised through TS14, resulting in the formation of the aldehyde (Min4). It can be observed that the opening of the epoxide is catalysed by the presence of the CO_2_ adduct. In the gas phase and isolated, the isomerisation of the epoxide exhibits a barrier of 52.6 kcal·mol^−1^. In the case of F2_NB_H_H, the barrier is reduced to 37.0 kcal·mol^−1^. CO_2_ later reacts with the aldehyde, forming the insertion product already observed in mechanism 1 (Min2). Passing through TS23, the product is released. Similar to mechanism 2, mechanism 3 contains eleven stationary points (Figure 5C). After the capture of CO_2_ by the FLP, the opening of the epoxide takes place along with the insertion reaction. The main difference from the previous two mechanisms is that a new intermediate (Min5) is stabilised, in which the oxygen of CO_2_ has attacked the electrophilic carbon of PO, and the oxygen atom of PO interacts with the LB. This mechanism is exclusive to phosphorus-containing FLPs, as nitrogen does not support this type of reactivity. Subsequently, the intermediate undergoes reorganization, leading to Min2.

Surprisingly, family 5, having phosphorus as the Lewis base, presents a different reactivity from the other families (Figure S3, Supporting Information File 1). Compounds F5_PB_H and F5_PB_CF3 react following mechanism 3 (Figure 5C), but the reaction proceeds directly from Min5 to Min3, with no Min2 observed. These two cases were then removed from the volcano plot analysis. The remaining two catalysts from family 5, namely, F5_NB_H and F5_NB_CF3, react according to mechanism 1 (Figure 5A, Figure S4, Supporting Information File 1). The energy matrix obtained can be found in Table S2 and Table S3 (Supporting Information File 1). It is interesting to observe that of the remaining 47 catalysts, 12 are not catalytically active, having their largest activation barrier greater than the 55.0 kcal·mol^−1^ previously reported for the uncatalysed reaction. Most of these belong to family 1.

#### Optimising catalyst selection

The optimal scaffolds and substituents for the studied reaction were identified through analysis using a volcano plot. This analysis requires that all catalytic cycles present the same number of steps. Because of varying numbers of steps between mechanism 1 and mechanisms 2 and 3, they had to be treated separately. The first group comprises catalysts that yield reactions following mechanism 1 (Figure 5A), characterised by nine stationary points. The second group consists of reactions with eleven stationary points, indicating that FLPs catalyse reactions following mechanisms 2 or 3 (Figure 5B,C). For each group, an analysis was performed using two volcano plots. The first plot aids in identifying the best families, which are then exclusively considered for the second volcano plot. The second plot helps to determine the most appropriate substituents to consider, thereby highlighting the optimal catalyst.

The first group of reactions, those following mechanism 1 (Figure 5A), comprises a total of twenty-two FLPs, accounting for 40% of the 55 catalysts considered. This group 1 includes FLPs from families 1, 3, 4, and 5. Sixteen compounds are based on an N/B pair, while the remaining six are P/B FLPs. Given the relative complexity of the mechanism studied, it was necessary to employ a 3D volcano plot using the energy span (δ*E*) and two energies of the system. Analysis of the correlations revealed that the most suitable combination of energies to consider involved the energy of pre-TS01 assembly, which is the non-covalent complex formed between the FLP and the CO_2_ molecule, and the energy of the intermediate Min2 (Figure 5). Correlating these parameters with the energy span yields an R^2^ value of 0.79, a mean absolute error (MAE) of 2.59, and a standard mean absolute percentage error (MAPE) of 0.35.

**Figure 6:**
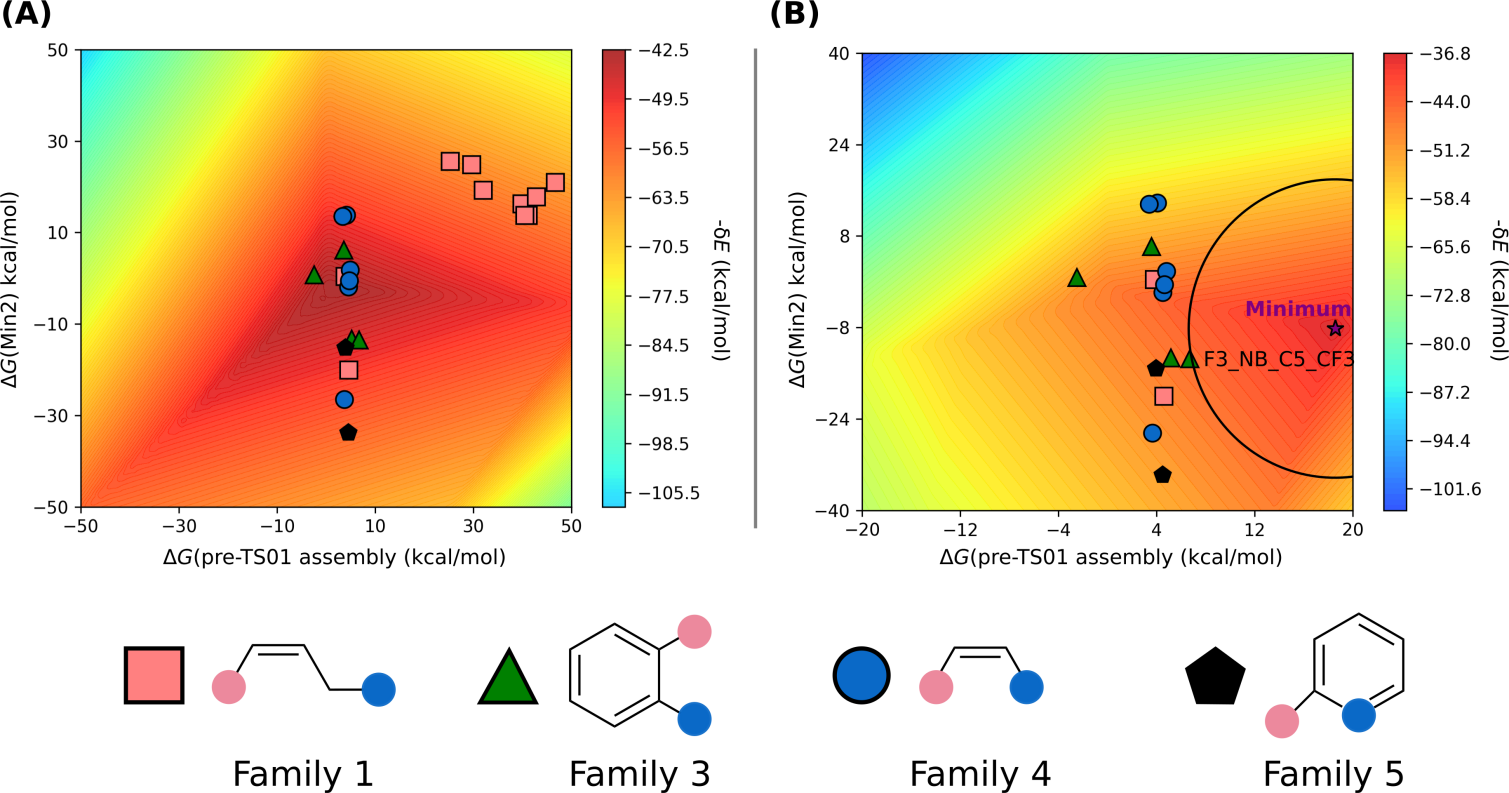
VOLCANO plot group 1. The free energies of pre-TS01 assembly and Min2 are considered for the correlation. (A) On the left, the compounds of families 1, 3, 4, and 5 were used for the plot. (B) On the right, only families 3, 4, and 5 are considered along with compounds F1_PB_Ph_H and F1_PB_Ph_CF3. In (B), a purple star was used to locate the minimum of the surface, along with a circle centred at the minimum to locate the closest systems. Also in (B), the names of the most effective catalysts are indicated in black.

As depicted in Figure 6A, families 3, 4, and 5 emerge as catalysts that catalyse the reaction most effectively. This aligns with previous findings that family 1 is not suitable for catalysing the reaction. However, compounds F1_PB_Ph_H and F1_PB_Ph_CF3 from family 1 are exceptions as they exhibit acceptable catalytic activity.

To identify the most suitable substituents, compounds of family 1 were excluded (except F1_PB_Ph_H and F1_PB_Ph_CF3), and a new volcano plot (Figure 6B) was generated. This plot employs the same axes as before (Δ*G*(pre-TS01 assembly) and Δ*G*(Min2)) and identifies a catalyst worthy of special consideration, that is, F3_NB_C5_CF3.

Additionally, it is observed that an efficient catalyst for this reaction should have an unstable pre-TS assembly, pre-TS01 assembly, (E1 > 0), and an intermediate Min2 with an energy close to 0 kcal·mol^−1^. Remarkably, among the most efficient catalysts within this group of FLPs those with a nitrogen LB stand out. This phenomenon could be attributed to the exceptional stability of the covalent adduct formed between phosphorus-based FLPs and CO_2_.

The second group comprises compounds that undergo reactions following mechanisms 2 or 3 (Figure 5). This group represents 60% of the 55 catalysts considered. This time, the set is richer in FLPs based on phosphorus, comprising 21 out of 29 compounds. It includes compounds from families 1, 2, 3, and 4. Similarly to the previous group, a 3D volcano plot was utilised. The same variables (energy of pre-TS01 assembly and Min2) were considered, which yielded a correlation with a R^2^ value of 0.71.

**Figure 7:**
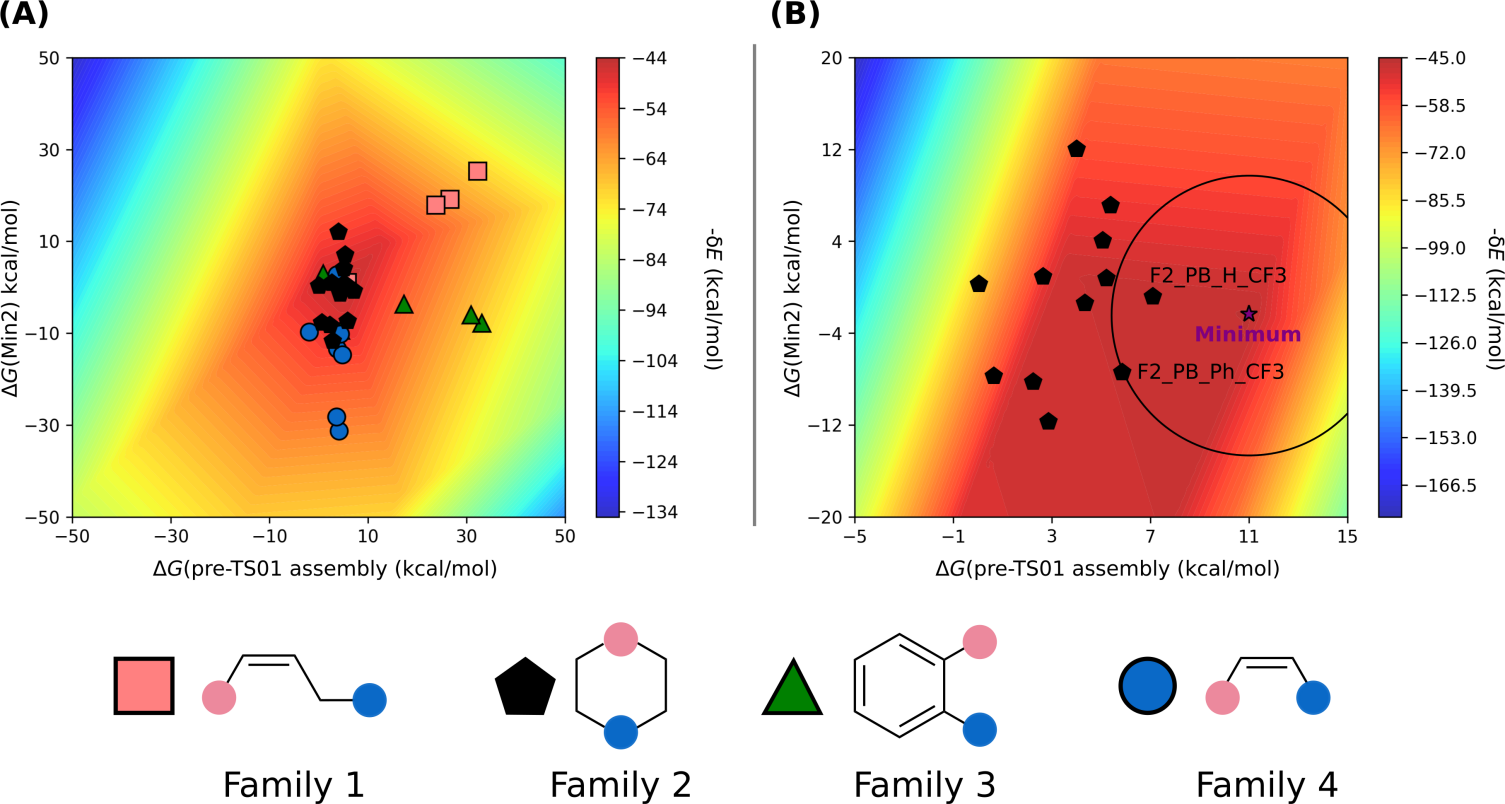
VOLCANO plot group 2. The free energies of pre-TS01 assembly and Min2 are considered for the correlation. (A) On the left, the compounds of families 1, 2, 3 and 4 are depicted. (B) On the right, only family 2 is considered. In (B) a purple star was used to locate the minimum of the surface, along with a circle centred at the minimum to locate the closest systems. Also in (B), the names of the most effective catalysts are indicated in black.

As depicted in Figure 7A, it is clear that the best family for this mechanism is family 2, followed by families 3 and 4. Family 1, similar to the previous group, exhibits the lowest catalytic activity. The low reactivity could be attributed to the masked character of this family. FLP monomers belonging to family 1 can be considered as masked FLPs [59–60], requiring breaking the LA–LB bond to achieve reactivity. Consequently, the pre-TS assembly formed between CO_2_ and the FLPs from family 1 are less stable than the pre-TS assembly between CO_2_ and the other FLP families, because of the absence of possible interactions between CO_2_ and LA or L). Furthermore, TS01, corresponding to the capture of CO_2_, is higher in energy because of the need for breaking the LA–LB bond.

By exclusively considering family 2 and using the energy of pre-TS01 assembly and product P4 for the second volcano plot (Figure 7B), it is observed that the best candidates are F2_PB_H_CF3 and F2_PB_Ph_CF3. Then, it can be concluded that the catalytic activity of the FLP is more efficient if the boron bears CF_3_ substituents. Thus, an acidic boron atom seems to increase the reactivity of the considered system. Concerning the LB, it appears that, as opposed to the first group of compounds, a phosphorus atom is more active than a nitrogen atom.

### Asymmetric catalysis

**Scheme 3:**
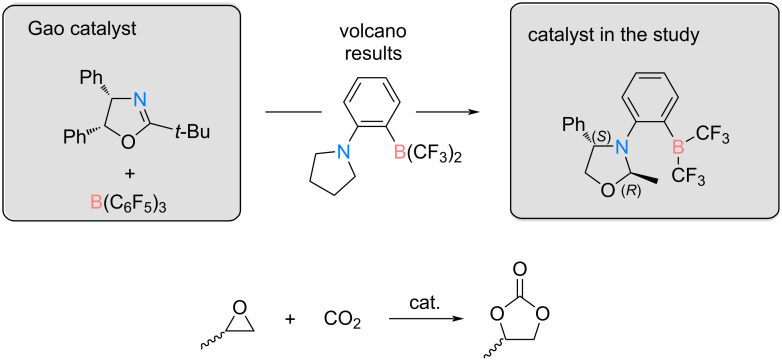
Asymmetric catalysis studied. On the left, the catalyst proposed by Gao et al. for the asymmetric hydrogenation of a ketone is shown [28]. On the right, the catalyst design inspired by the Gao catalyst and the volcano plot results is shown. At the bottom the reaction under study is presented.

After examining the volcano results, we looked into the literature to explore examples of asymmetric FLPs. This exploration revealed three main types, namely, intramolecular chiral FLPs, intermolecular FLPs composed of a chiral acid and an achiral base, and intermolecular FLPs comprising an achiral acid and a chiral base [61–62]. One study reported a reaction involving the asymmetric reduction of ketones using an achiral borane, denoted as B(*p*-HC_6_F_4_)_3_, paired with a chiral oxazoline, as depicted in Scheme 3 [28]. Remarkably, in this study, these FLPs demonstrated the capability to achieve high conversion rates and enantiomeric excess.

Following the volcano plot analysis presented in the previous section, F3_NB_C5_CF3 emerged as one of the top FLP catalysts under study. This catalyst, adhering to mechanism 1, incorporates a CF_3_ group on the boron atom, serving as a simplified version of the B(*p*-HC_6_F_4_)_3_. Notably, the nitrogen in this FLP is situated within a five-membered ring. Using this structural insight, an asymmetric catalyst was subsequently designed by strategically modifying the pyrrolidine substituent (C5 in Figure 4) based on the most efficient FLP.

The coupling reaction proposed in Scheme 3 was studied. In order to minimise the computational costs associated with the study, the asymmetric catalyst was obtained by removing a phenyl group and exchanging the *t*-Bu group with a methyl group in the catalyst of Gao [28]. It appears that the capture of CO_2_ by the catalyst is barrierless and results in the formation of an adduct with a relative free energy of 0.7 kcal·mol^−1^. Thus, the evaluation of the stereoselectivity of the designed catalyst was conducted by only studying the steps after the capture of CO_2_ by the catalyst.

The reaction occurs in two steps (Table 1). Initially, a pre-TS assembly, with the PO compound positioned 2.67 Å from the CO_2_ carbon is formed. Overcoming a TS, an intermediate is generated. In this intermediate, the distance between PO and the CO_2_ carbon decreases to 1.61 Å from the initial 2.67 Å, and the interaction between nitrogen and the CO_2_ carbon weakens. The intermediate is highly energetic and closely positioned to the TS. In the case of the (*R*) mechanism, the intermediate is slightly higher in energy than the TS, potentially because of methodological error. The intermediate further reacts with the activated CO_2_ to generate the corresponding product. As can be observed in Table 1, the mechanism leading to the (*S*) product presents an asymmetric TS, 1.2 kcal·mol^−1^ higher in energy than that of the (*R*) mechanism. Thus, the asymmetric catalyst enables to generate an enantiomeric excess of 95% with the (*R*) product being the most abundant product.

**Table 1:** Free energy reaction profile of the asymmetric coupling between propylene oxide and CO_2_ catalysed by the catalyst depicted in Scheme 3. The energies are reported in kcal·mol^−1^, and the 0.0 energy was set to be the FLP–CO_2_ adduct and the isolated propylene oxide.

Enantiomer	Pre-TS	TS1	Int.	Asymmetric TS	Product

*R*	9.4	27.6	27.9	30.9	−14.2
*S*	8.9	25.0	24.5	32.1	−13.4

**Figure 8:**
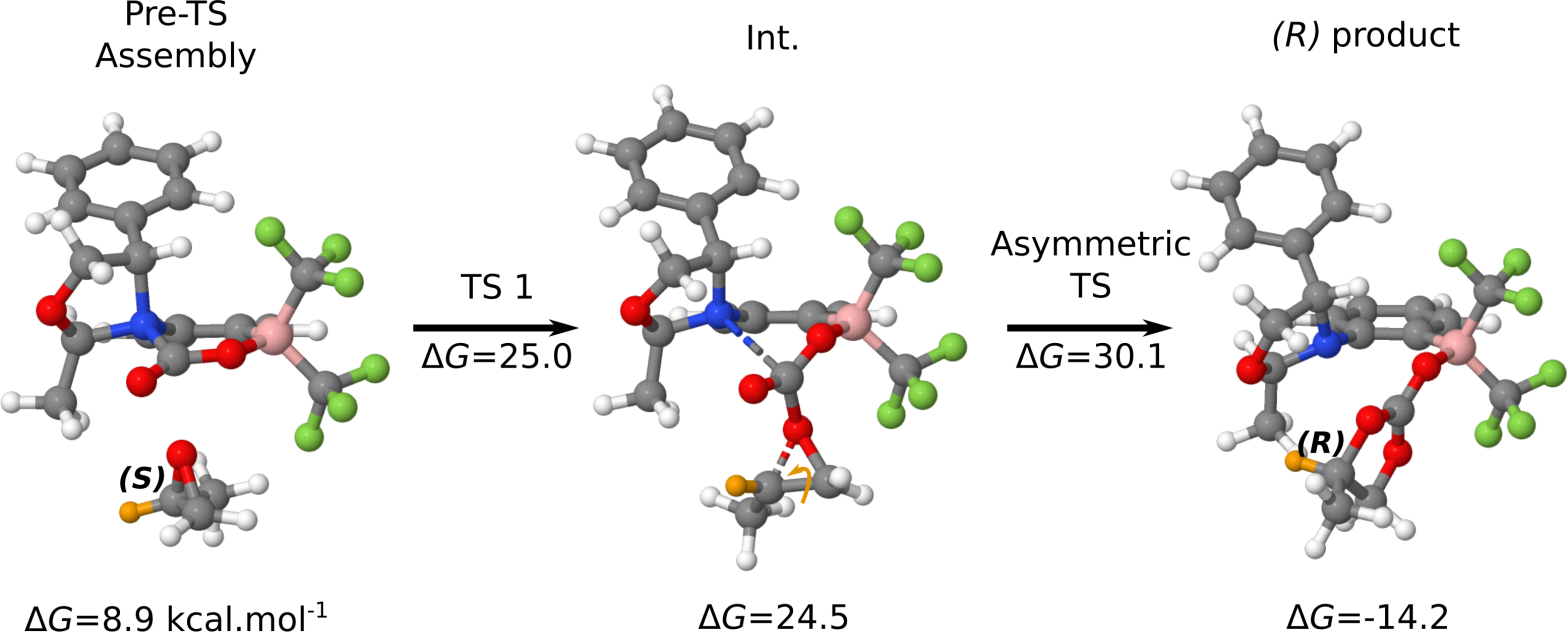
Catalysed reaction between the (S)-enantiomer of propylene oxide and CO_2_ resulting in the formation of the (*R*)-product. In orange, the hydrogen atom that illustrates the stereochemistry inversion. The free energies are given in kcal·mol^−1^

**Figure 9:**
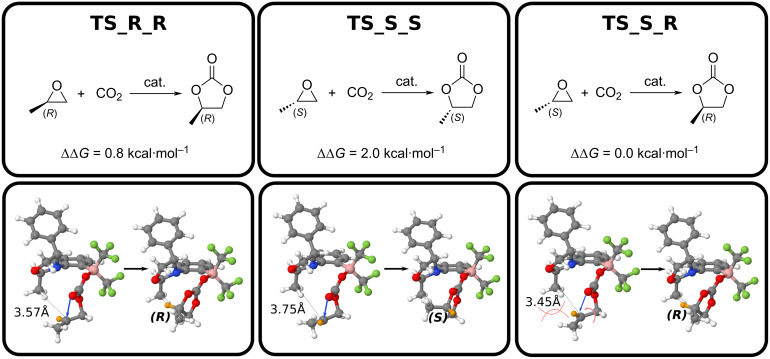
Schemes of the different asymmetric reactions observed. Hydrogen capable of rotation is marked in orange, influencing the stereochemistry at the TS.

Surprisingly, a transition state connecting the (*S*) epoxide with the (*R*) product was identified (Figure 8). Even more intriguingly, this new TS (TS_S_R in ), verified by the IRC calculation (Figure S5, Supporting Information File 1) is the most stable TS located (Figure 9).

In this TS, the epoxy ring opens (Figure 9). Because of a shorter C–C distance between the CH_3_ group in the catalyst and the epoxy carbon atom (3.45 Å vs 3.75 Å in TS_S_S), a steric clash between the two methyl groups occurs (Figure 9). This results in an inversion of stereochemistry via rotation of the epoxy C–C bond, leading to the formation of the (*R*) product. As two TSs now yield the same product, it is necessary to recalculate the %ee, but this time using an effective rate constant *k*_eff_ (Equation 3). In doing so, a small increase in enantioselectivity is observed, with now a (*R*) enantiomeric excess of 96%ee. The designed catalyst enables the generation of an almost enantiomerically pure product from a racemic mixture.

## Conclusion

Carbon capture and utilisation technologies represent a promising avenue for addressing increasing atmospheric carbon dioxide levels. The reaction involving the insertion of CO_2_ into epoxides to form cyclic carbonates is a key focus within this domain. Despite extensive exploration, the stereochemical aspects of this reaction have been surprisingly underexplored, especially in the context of racemic epoxide mixtures commonly encountered in practice.

This study introduces an innovative in silico design strategy for asymmetric frustrated Lewis pairs tailored specifically to control the stereochemistry of the CO_2_ insertion reaction. Computational evaluations of four distinct FLP scaffolds, incorporating various Lewis acids, Lewis bases, and substituents, identify the most promising catalyst candidates through volcano plot analysis. The volcano plot analysis reveals that the best candidate is F3_NB_C5_CF3, which is the catalyst based on the 2-borylbenzenamine scaffold, with a pyrrolidine substituent on the nitrogen atom and CF_3_ substituents on the boron.

Through strategic modification of the Lewis base substituents, a stereoselective catalyst was engineered to produce a single enantiomer preferentially from both enantiomers of the epoxide substrate. An enantiomeric excess of 95%ee was initially achieved, with the predominant (*R*) enantiomer. Enhanced selectivity was subsequently observed through additional transition states, resulting in a remarkable 96%ee yielded by the catalyst.

## Supporting Information

Supporting information features geometries of the different stationary points optimised as well as figures and tables mentioned in the main text. The outputs of the calculations presented can be found at the following link: https://doi.org/10.5281/zenodo.

File 1 Supporting figures and tables.

## Data Availability

The data generated and analyzed during this study are openly available in Zenodo at https://doi.org/10.5281/zenodo.12633864.
