# Supplementary material for: Computational design for enantioselective CO2 capture: asymmetric frustrated Lewis pairs in epoxide transformations
Source: Beilstein J Org Chem. 2024 Oct 22;20:2668–81. doi: 10.3762/bjoc.20.224 (PMC11514440; doi:10.3762/bjoc.20.224)
Supplement: File 1 — Supporting figures and tables. [file Beilstein_J_Org_Chem-20-2668-s001.pdf]

BEILSTEIN JOURNAL OF ORGANIC CHEMISTRY

Supporting Information

for

Computational design for enantioselective CO_2 capture: asymmetric frustrated Lewis pairs in epoxide transformations

Maxime Ferrer, Iñigo Iribarren, Tim Renningholtz, Ibon Alkorta and Cristina Trujillo

Beilstein J. Org. Chem. **2024,** *20,* 2668–2681. doi:10.3762/bjoc.20.224

Supporting figures and tables

License and Terms: This is a supporting information file under the terms of the Creative Commons Attribution License (https://creativecommons.org/licenses/by/4.0). Please note that the reuse, redistribution and reproduction in particular requires that the author(s) and source are credited and that individual graphics may be subject to special legal provisions.
The license is subject to the *Beilstein Journal of Organic Chemistry* terms and conditions: (https://www.beilstein-journals.org/bjoc/terms)

SI Online

The outputs of the calculations presented can be found at the following link:

https://doi.org/10.5281/zenodo.12633864

List of Figures

S1 Free-energy profiles of the capture of CO_2 (pink) and epoxide (E) (dark) by FLP. . . S3

S2 Free energy of the adduct formed between CO_2 and the different FLP scaffolds. . . . S4

S3 FIA and PA of the LA and LB respectively in kJ·mol^{-1} for the different scaffolds and substituents. S5

S4 Reaction profile for family 5. . . . . S5

S5 IRC of the asymmetric (*R*) TS (left) and (*S*) TS (right). The zero energy was set to be the sum of the CO_2 adduct energy plus the energy of the isolated epoxide. S8

S6 Free energy profile of the uncatalysed reaction between CO_2 and ethylene oxide. The zero was set to be the sum between the energy of CO_2 and isolated ethylene oxide. . . S9

List of Tables

S1 Energies of the first TS (FLP/E) for the capture of different epoxide by a FLP. S4

S2 Energy matrix for families 1 and 2. The energies are given in kcal·mol^{-1}. A grey cell means that the stationary point does not exist for the considered catalyzed reaction. S6

S3 Energy matrix for families 1 and 2. The energies are given in kcal·mol^{-1}. A grey cell means that the stationary point does not exist for the considered catalyzed reaction. S7

S4 NBO Lewis base and acid charges, distance between the Lewis acid and the Lewis base, minimum and maximum of the molecular electrostatic potential associated with the Lewis base and the Lewis acid, respectively, density at the BCP, and Laplacian at the BCP for the masked FLPs. S10

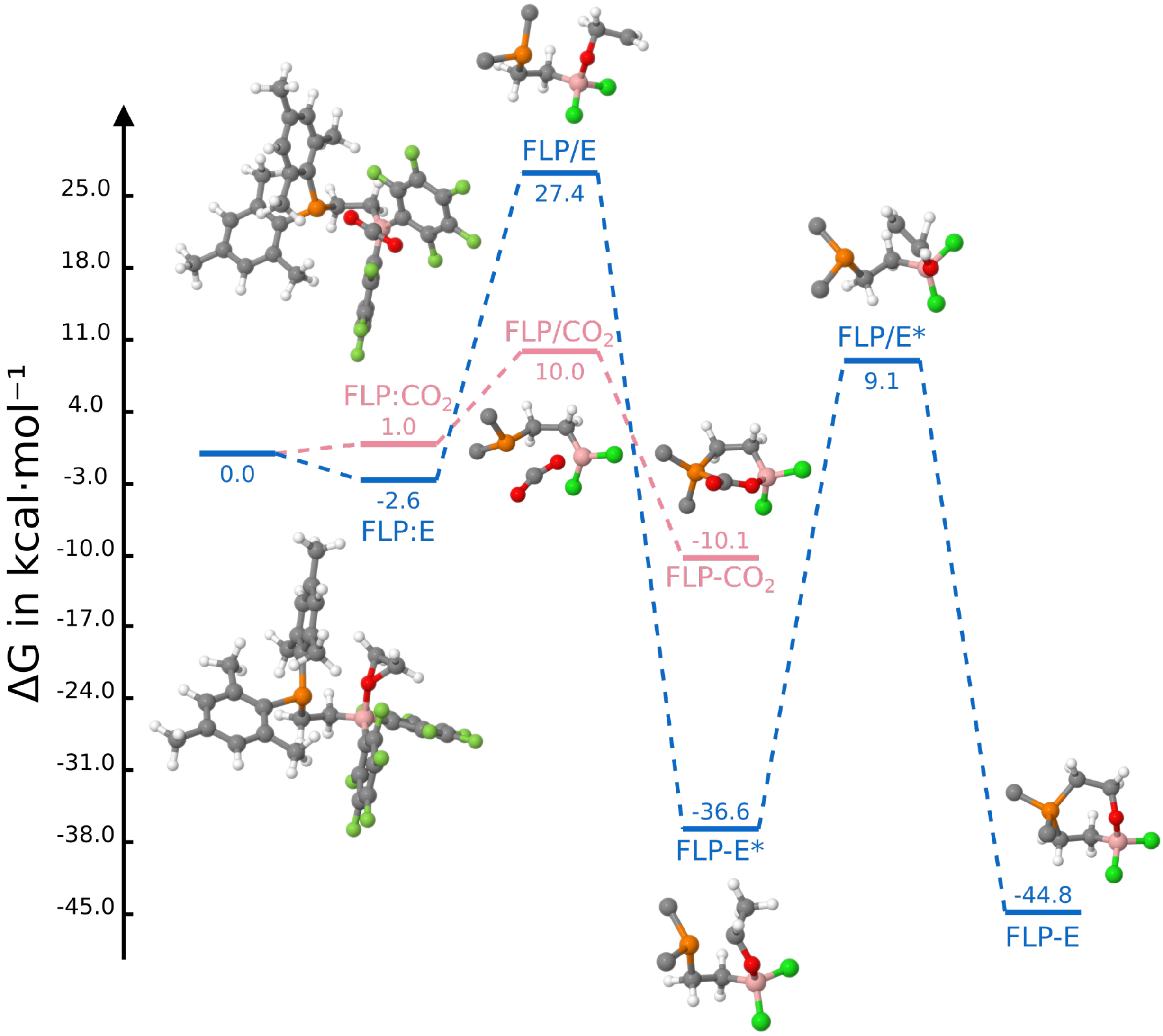

Figure S1: Free-energy profiles of the capture of CO_2 (pink) and epoxide (E) (dark) by FLP.

Table S1: Energies of the first TS (FLP/E) for the capture of different epoxide by a FLP.

Substituent on the epoxide	E(TS) (Hartree)	Erel(TS) (kJ/mol)
H	−2751.723076	63.2
CH_3	−2979.688861	69.3
Ph	−3171.518481	69.6
t-Bu	−3097.689750	74.7

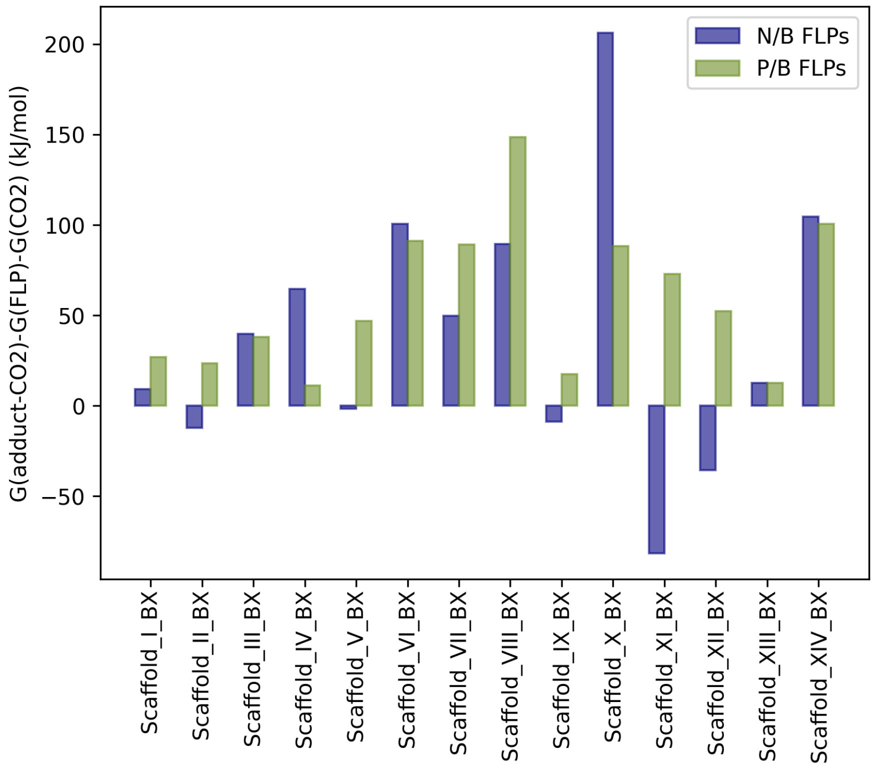

Figure S2: Free energy of the adduct formed between CO_2 and the different FLP scaffolds.

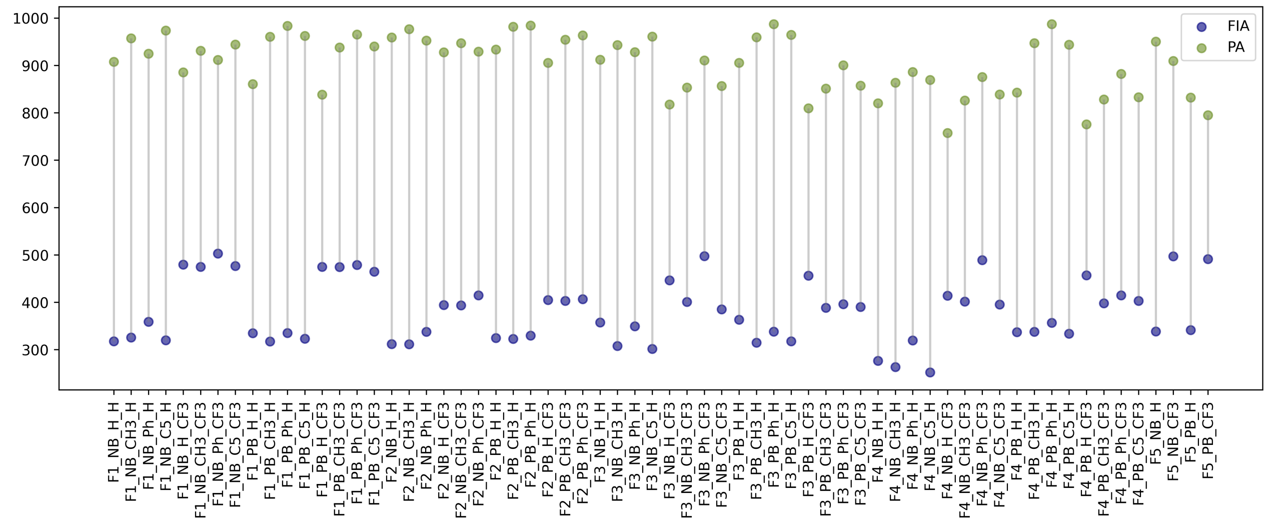

Figure S3: FIA and PA of the LA and LB respectively in kJ·mol^{-1} for the different scaffolds and substituents.

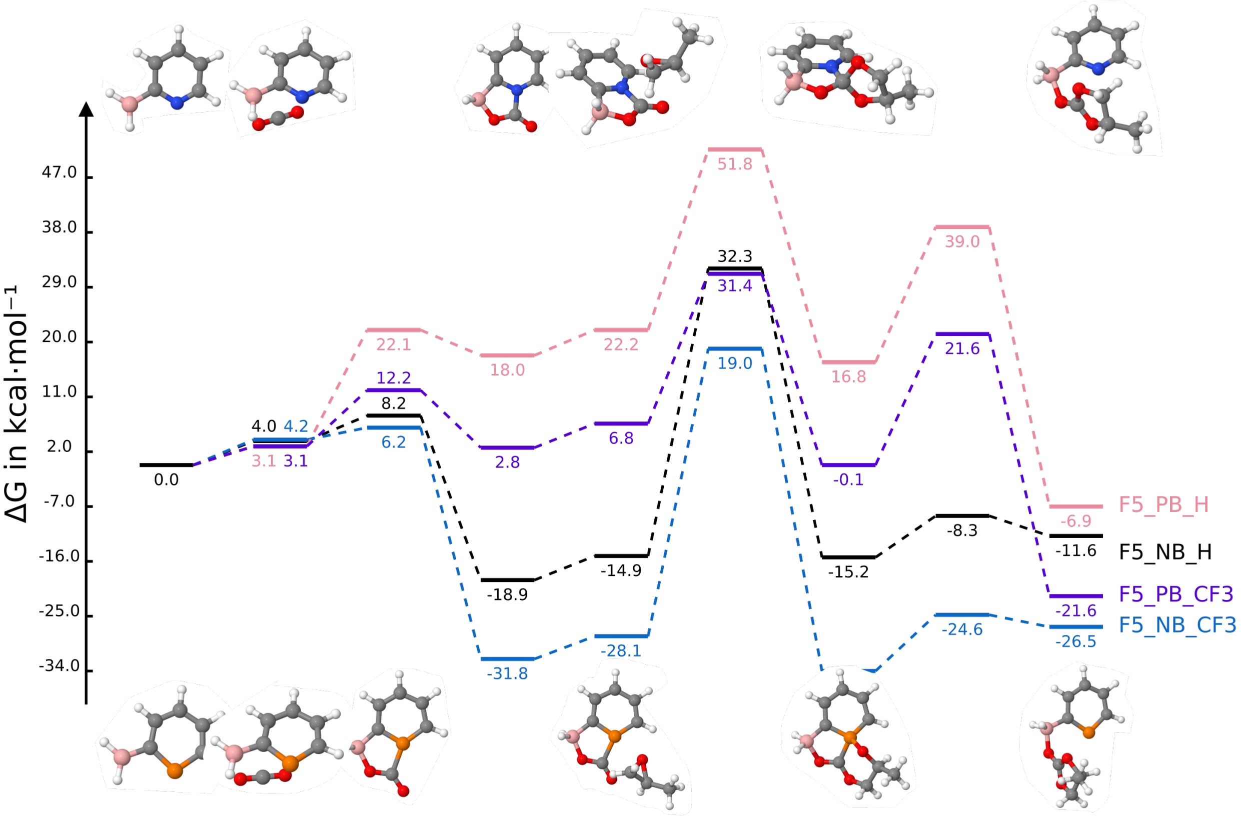

Figure S4: Reaction profile for family 5.

Table S2: Energy matrix for families 1 and 2. The energies are given in kcal·mol^{-1}. A grey cell means that the stationary point does not exist for the considered catalyzed reaction.

Family	LB	Substituent	FLP	PTS01	TS01	P1	PTS12/PTS13	TS12/TS13	P2	TS23	P3	TS34	P4	PROD
1	N	H_H	0	29.7	36.1	25.1	29.6	78.8			24.8	24.8	20.4	−8.2
		CH3_H	0	5.8	12.4	2.9	6.9	53.9	−16.0	49.2	1.0	0.9	−3.9	−8.2
		Ph_H	0	11.7	29.1	28.9	33.0	47.8					2.8	−8.2
		C5_H	0	32.2	38.0	25.8	30.1	76.1	7.3	72.9	25.3	25.7	22.9	−8.2
		H_CF3	0	46.7	49.1	27.0	31.4	74.8			20.9	23.2	20.7	−8.2
		CH3_CF3	0	39.9	41.0	21.5	26.2	62.2			16.3	18.8	16.6	−8.2
		Ph_CF3	0	19.4	23.7	18.4	23.3	30.6					−6.8	−8.2
		C5_CF3	0	42.9	45.2	22.2	25.6	66.2			17.8	19.8	18.2	−8.2
1	P	H_H	0	25.3	33.7	25.3	28.1	25.6			25.6	25.6	8.3	−8.2
		CH3_H	0	26.6	34.3	19.0	23.2	19.1	0.6	61.5	19.1	27.9	18.0	−8.2
		Ph_H	0	3.8	13.5	1.6	4.6	53.6			0.4	4.9	−6.8	−8.2
		C5_H	0	23.7	32.7	18.3	21.6	17.9	−1.2	59.9	17.9	26.7	16.3	−8.2
		H_CF3	0	32.0	41.5	23.6	24.6	19.2			19.2	13.5	10.2	−8.2
		CH3_CF3	0	41.1	47.0	17.8	19.6	68.7			13.7	27.4	22.6	−8.2
		Ph_CF3	0	4.6	10.2	−15.6	−12.7	35.8			−20.1	−11.0	−15.5	−8.2
		C5_CF3	0	40.4	46.5	16.9	19.4	68.8			13.8	27.2	20.9	−8.2
2	N	H_H	0	2.6	9.2	−1.6	3.5	36.7	−22.7	44.1	0.9	4.9	−3.9	−8.2
		CH3_H	0	0.0	8.0	−3.5	1.6	47.3	−24.3	42.1	0.3	2.8	−5.5	−8.2
		Ph_H	0	4.0	17.0	11.1	15.2	60.8	−10.4	56.2	12.0	13.0	−1.5	−8.2
		H_CF3	0	2.2	9.7	−8.4	−3.3	42.8	−29.8	37.2	−8.3	−3.2	−9.0	−8.2
		CH3_CF3	0	0.7	6.2	−9.6	−4.3	41.2	−30.4	36.7	−7.7	−4.5	−6.4	−8.2
		Ph_CF3h	0	5.1	15.1	5.2	9.3	54.6	−16.6	51.4	4.1	5.5	−6.4	−8.2
2	P	H_H	0	5.4	12.8	5.6	9.8	53.4	14.0	25.5	7.1	14.1	−0.2	−8.2
		CH3_H	0	4.3	8.6	−3.9	0.2	44.7	12.9	19.9	−1.4	9.9	−1.6	−8.2
		Ph_H	0	5.2	10.5	−0.7	3.7	47.8	18.6	21.5	0.8	10.1	−0.8	−8.2
		H_CF3	0	7.1	16.8	−1.2	3.7	45.6	6.8	19.9	−0.8	6.9	−2.8	−8.2
		CH3_CF3	0	2.9	9.5	−12.3	−8.2	35.9	3.8	12.1	−11.7	1.0	−6.1	−8.2
		Ph_CF3	0	5.8	14.5	−6.6	−2.4	41.4	11.3	15.9	−7.4	3.7	−3.2	−8.2

Table S3: Energy matrix for families 1 and 2. The energies are given in kcal·mol^{-1}. A grey cell means that the stationary point does not exist for the considered catalyzed reaction.

FFamily	LB	Substituent	FLP	PTS01	TS01	P1	PTS12/PTS13	TS12/TS13	P2	TS23	P3	TS34	P4	PROD
3	N	H_H	0	−2.5	10.9	3.6	4.4	54.5			0.8	1.6	−9.3	−8.2
		C5_H	0	3.6	16.5	6.7	10.6	34.6			6.1	7.3	−3.4	−8.2
		H_CF3	0	5.2	10.7	−8.0	−8.5	37.5			−13.4	−12.9	−19.0	−8.2
		C5_CF3	0	6.7	16.7	−7.7	−2.8	37.3			−13.5	−7.9	−11.1	−8.2
3	P	H_H	0	0.9	14.8	3.8	7.8	48.1	9.4	25.0	2.9	4.3	−10.0	−8.2
		CH3_H	0	3.6	11.3	−11.4	−8.5	37.0	−34.7	28.8	−10.9	−1.0	−5.2	−8.2
		C5_H	0	4.5	11.1	−10.0	−7.8	37.7	−33.3	30.2	−9.4	0.3	−4.4	−8.2
		H_CF3	0	17.3	23.3	1.5	3.0	43.7	5.6	89.2	−3.7	0.4	−5.6	−8.2
		CH3_CF3	0	33.1	34.6	−3.4	0.7	44.6	−26.2	36.8	−7.8	8.5	9.7	−8.2
		C5_CF3N	0	30.9	34.8	−3.1	0.1	43.8	−26.3	37.2	−6.0	8.6	10.9	−8.2
4	N	H_H	0	4.1	24.5	16.0	15.9	67.2			13.8	14.0	3.8	−8.2
		Ph_H	0	3.7	26.2	24.5	30.1	45.8					−1.5	−8.2
		C5_H	0	3.4	24.8	14.3	19.2	43.3			13.5	15.8	−4.5	−8.2
		H_CF3	0	4.8	23.4	7.0	6.5	52.7			1.8	1.8	−6.4	−8.2
		CH3_CF3	0	4.5	26.5	2.9	7.9	49.2			−1.9	0.9	−1.9	−8.2
		Ph_CF3	0	−8.3	9.6	1.6	7.2	20.7					−18.5	−8.2
		C5_CF3	0	4.6	26.4	4.3	9.4	51.6			−0.5	4.0	−0.3	−8.2
4	P	H_H	0	3.7	17.8	3.3	6.6	50.3	10.9	27.6	2.7	4.6	−7.1	−8.2
		CH3_H	0	3.8	12.2	−13.6	−9.6	36.2	−32.1	30.1	−13.4	−2.9	−5.5	−8.2
		Ph_H	0	−2.0	5.9	−8.7	−3.5	40.6	−27.0	35.5	−9.8	−3.3	−7.5	−8.2
		C5_H	0	4.3	14.1	−11.6	−7.0	37.8	−30.1	32.7	−10.1	−1.0	−4.6	−8.2
		H_CF3	0	4.8	10.8	−10.7	−7.5	32.6	−3.0	12.0	−14.7	−10.8	−18.3	−8.2
		CH3_CF3	0	4.1	8.4	−28.3	−23.7	20.5	−47.3	14.2	−31.3	−18.2	−16.9	−8.2
		Ph_CF3	0	3.7	10.3	−23.3	−19.1	20.2			−26.5	−17.0	−17.0	−8.2
		C5_CF3	0	3.6	10.0	−25.5	−22.6	22.2	−45.1	16.8	−28.2	−15.8	−15.0	−8.2

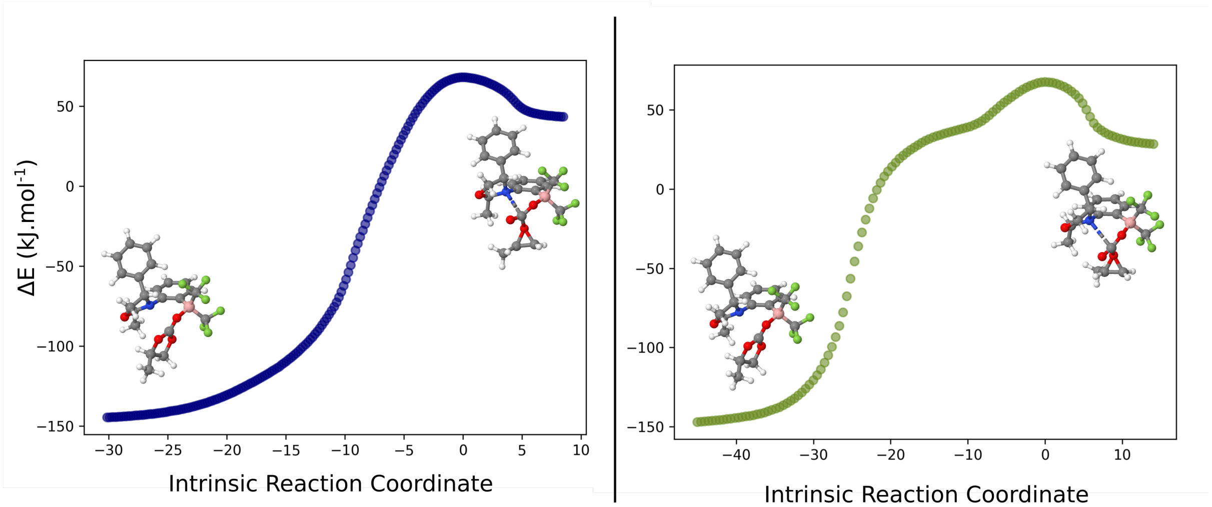

Figure S5: IRC of the asymmetric (*R*) TS (left) and (*S*) TS (right). The zero energy was set to be the sum of the CO_2 adduct energy plus the energy of the isolated epoxide.

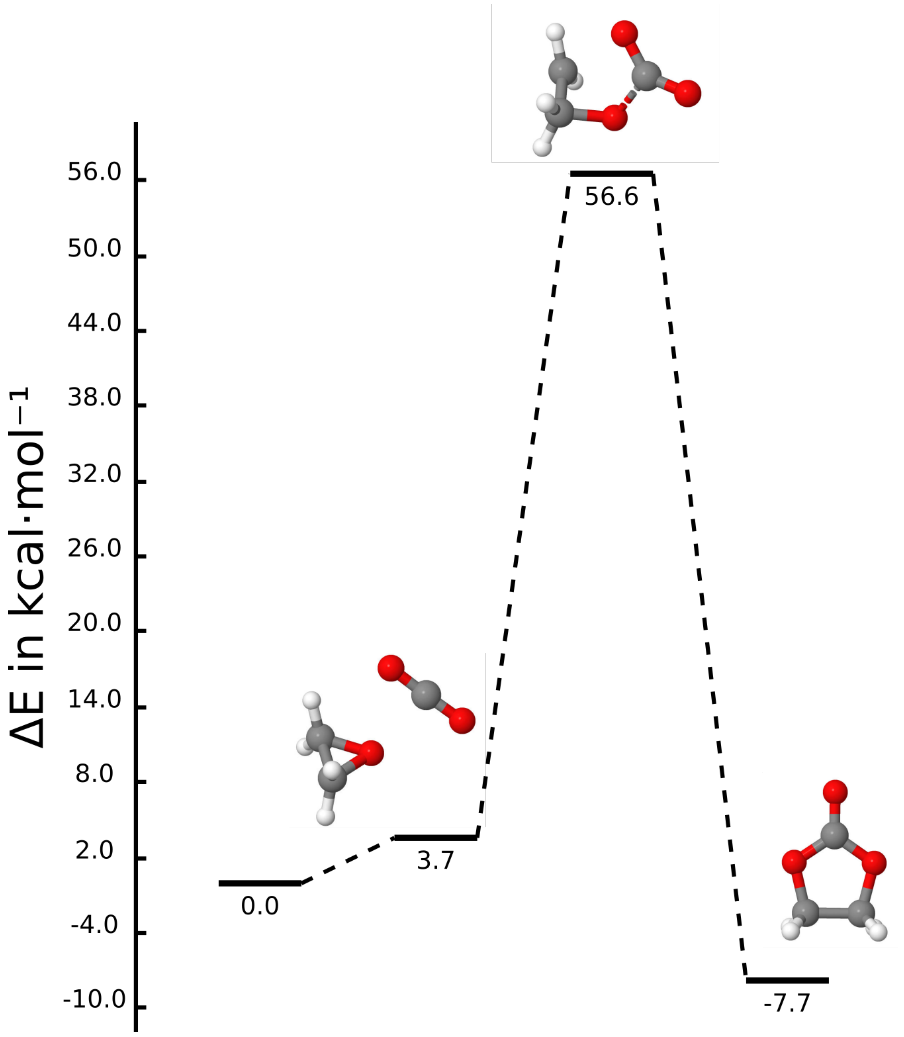

Figure S6: Free energy profile of the uncatalysed reaction between CO_2 and ethylene oxide. The zero was set to be the sum between the energy of CO_2 and isolated ethylene oxide.

Table S4: NBO Lewis base and acid charges, distance between the Lewis acid and the Lewis base, minimum and maximum of the molecular electrostatic potential associated with the Lewis base and the Lewis acid, respectively, density at the BCP, and Laplacian at the BCP for the masked FLPs.

			Classical FLP conformation				
			LB charge	LA charge	d(LA-LB)	Minimum LB	Maximum LA
			a.u.	a.u.	Å	$kJ\cdot mol^{-1}$	$kJ\cdot mol^{-1}$
1	N	H_H	−0.81008	0.38456	4.355964	−155.6	46.7
		CH3_H	−0.40771	0.38175	4.436370	−119.9	52.7
		Ph_H	−0.39681	0.38094	4.383981	−11.5	81.1
		C5_H	−0.40542	0.38325	4.358531	−124.4	49.6
		H_CF3	−0.81382	0.61428	4.643562	−95.8	146.5
		CH3_CF3	−0.41056	0.61712	4.644180	−69.3	146.5
		Ph_CF3	−0.40524	0.61574	4.667826		164.3
		C5−CF3	−0.41051	0.61605	4.601636	−85.0	149.9
	P	H_H	0.30791	0.37773	4.044480	−92.4	38.3
		CH3_H	0.80972	0.37447	3.945418	−125.7	37.2
		Ph_H	0.83769	0.37489	3.955863	−113.4	18.6
		C5_H	0.76202	0.37591	4.054490	−128.1	36.4
		H_CF3	0.32777	0.61005	4.261709	−72.2	124.9
		CH3_CF3	0.82737	0.60648	4.153283	−104.2	120.7
		Ph_CF3	0.85546	0.60727	4.123698	−98.7	125.4
		C5−CF3	0.77797	0.60202	4.287729	−104.7	101.3
2	N	H_H	−0.60138	0.66082	2.925893	−158.7	116.8
		H_CH3	−0.41876	0.66635	2.931085	−141.7	114.4
		H_Ph	−0.38482	0.66771	2.864116	−92.9	137.8
		CF3_H	−0.59760	0.80957	2.899121	−131	147
		CF3_CH3	−0.41478	0.81765	2.905267	−114.4	146.7
		CF3_Ph	−0.38427	0.81797	2.838805	−69.5	167.5
	P	H_H	0.53161	0.66932	3.258438	−126.8	126.5
		H_CH3	0.76604	0.67121	3.279519	−141.2	120.7
		H_Ph	0.79475	0.67395	3.272599	−130.7	121.5
		CF3_H	0.54112	0.82405	3.239329	−104.2	157.2
		CF3_CH3	0.76270	0.82729	3.264229	−119.4	152.2
		CF3_Ph	0.80118	0.83120	3.250620	−109.7	150.7
3	N	H_C4N	−0.35483	0.33631	3.135547		−12.8
		CF3_H	−0.73083	0.58521	3.045139		100
		CF3_C4N	−0.33049	0.58135	3.129673		104.7
	P	H_CH3	0.80616	0.40527	3.215669	−132.5	43.8
		H_C4N	0.77544	0.40426	3.207660	−130.7	40.1

			Classical FLP conformation				
			LB charge	LA charge	d(LA-LB)	Minimum LB	Maximum LA
			a.u.	a.u.	Å	kJ·mol^{-1}	kJ·mol^{-1}
4	N	H_H	−0.74016	0.28853	3.053427		−33.3
		H_Ph	−0.39333	0.30760	3.275301		−32.2
		H_C4N	−0.34449	0.25448	3.265651		−70.6
		CF3_H	−0.69889	0.48755	3.200662		55.1
		CF3_CH3	−0.30291	0.43359	3.326664		−0.2
		CF3_C4N	−0.30534	0.43818	3.313716		−4.9
	P	H_H	0.31543	0.38333	3.409102	−66.1	63.7
		H_CH3	0.79337	0.37372	3.378810	−109.4	42
		H_Ph	0.83210	0.38672	3.326559	−98.4	48.5
		H_C4N	0.77514	0.35847	3.373464	−104.2	29.4
		CF3_H	0.36719	0.60176	3.612198	−26.7	141.2
		CF3_CH3	0.86524	0.58040	3.656968	−63.7	86.3
		CF3_Ph	0.80926	0.45044	3.420171	−74.5	
		CF3_C4N	0.84542	0.56912	3.624319	−62.4	77.4
5	N	H	−0.37143	0.43369	2.492780	−182.7	77.4
		CF3	−0.35615	0.68147	2.446942	−145.9	165.4
	P	H	0.74568	0.40793	2.797578	−91.3	63.5
		CF3	0.79115	0.66459	2.807091		148.6

			Masked-FLP conformation				
			LB charge	LA charge	d(LA-LB)	ρ(BCP)	$\nabla 2(\rho)$
			a.u.	a.u.	Å	a.u.	a.u.
1	N	H_H	−0.64422	−0.01685	1.64616	0.1173	0.2746
		CH3_H	−0.32661	0.01102	1.66082	0.1166	0.2508
		Ph_H	−0.37467	0.01687	1.6971	0.1053	0.2335
		C5_H	−0.33066	0.01418	1.64614	0.1199	0.2691
		H_CF3	−0.64296	0.21906	1.62231	0.1289	0.2482
		CH3_CF3	−0.32545	0.27494	1.6528	0.1252	0.1716
		Ph_CF3	−0.37534	0.30561	1.70849	0.11	0.1212
		C5-CF3	−0.33351	0.27612	1.64137	0.1278	0.1859
	P	H_H	0.78724	−0.45036	1.97349	0.1104	−0.1183
		CH3_H	1.34793	−0.50055	1.95316	0.1205	−0.1642
		Ph_H	1.33935	−0.47381	1.96624	0.1155	−0.1433
		C5_H	1.30755	−0.48802	1.95831	0.1179	−0.1553
		H_CF3	0.81169	−0.18527	1.97725	0.1187	−0.208
		CH3_CF3	1.37262	−0.22430	1.97623	0.1241	−0.2436
		Ph_CF3	1.37841	−0.19722	1.98737	0.1202	−0.2277
		C5-CF3	1.33138	−0.21130	1.97683	0.1227	−0.2364
3	N	H_H	−0.67773	0.06979	1.725825	0.0979	0.1824
	P	H_H	0.70160	−0.24394	2.062502	0.0914	−0.0952
		CF3_H	0.77134	−0.03683	2.021633	0.1078	−0.1742
		CF3_CH3	1.32809	−0.10775	2.006233	0.1166	−0.2094
		CF3_C4N	1.29228	−0.09486	2.005830	0.1156	−0.2043
4	N	CF3_Ph	−0.39807	0.34359	1.755158	0.0994	0.0701
